# Microbial Composition of the Human Nasopharynx Varies According to Influenza Virus Type and Vaccination Status

**DOI:** 10.1128/mBio.01296-19

**Published:** 2019-07-02

**Authors:** Tao Ding, Timothy Song, Bin Zhou, Adam Geber, Yixuan Ma, Lingdi Zhang, Michelle Volk, Shashi N. Kapadia, Stephen G. Jenkins, Mirella Salvatore, Elodie Ghedin

**Affiliations:** a Center for Genomics & Systems Biology, Department of Biology, New York University, New York, New York, USA; b Department of Medicine, Weill Cornell Medical College, New York, New York, USA; c Department of Pathology, Weill Cornell Medical College, New York, New York, USA; d Department of Epidemiology, College of Global Public Health, New York University, New York, New York, USA; University of Maryland School of Medicine; University of California, Davis; University of Michigan Medical School

**Keywords:** 16S RNA sequencing, influenza virus, microbiome, vaccination

## Abstract

Factors that contribute to enhanced susceptibility to severe bacterial disease after influenza virus infection are not well defined but likely include the microbiome of the respiratory tract. Vaccination against influenza, while having variable effectiveness, could also play a role in microbial community stability. We collected nasopharyngeal samples from 215 individuals infected with influenza A/H3N2 or influenza B virus and profiled the microbiota by target sequencing of the 16S rRNA gene. We identified signature taxonomic groups by performing linear discriminant analysis and effective size comparisons (LEfSe) and defined bacterial community types using Dirichlet multinomial mixture (DMM) models. Influenza infection was shown to be significantly associated with microbial composition of the nasopharynx according to the virus type and the vaccination status of the patient. We identified four microbial community types across the combined cohort of influenza patients and healthy individuals with one community type most representative of the influenza virus-infected group. We also identified microbial taxa for which relative abundance was significantly higher in the unvaccinated elderly group; these taxa include species known to be associated with pneumonia.

## Abstract (precis)

Our results suggest that there is a significant association between the composition of the microbiota in the nasopharynx and the influenza virus type causing the infection. We observe that vaccination status, especially in more senior individuals, also has an association with the microbial community profile. This indicates that vaccination against influenza, even when ineffective to prevent disease, could play a role in controlling secondary bacterial complications.

## INTRODUCTION

Influenza virus is the major cause of severe viral respiratory infection in adults, resulting in more than 200,000 hospitalizations and 30,000 to 50,000 deaths each year in the United States (1). A critical factor in influenza virus-associated morbidity and mortality is the increased susceptibility of infected individuals to bacterial pneumonia, a common complication of influenza pandemics (2, 3) and epidemics (4). Epidemiological studies have shown that despite circulating in humans since 1968, seasonal H3N2 influenza outbreaks are associated with increased clinical severity, including excess respiratory mortality and excess pneumonia and influenza hospitalizations (5). During the 2014–2015 season, the antigenically drifted H3N2 influenza virus caused major outbreaks globally, resulting in increased pneumonia- and influenza-associated mortality (http://www.cdc.gov/flu/weekly/weeklyarchives2014-2015/week2.htm#S2) (6).

Although annual trivalent influenza vaccines are available and widely received, vaccine effectiveness can be limited. Vaccines are less effective in the elderly, a population that is particularly vulnerable to influenza infections and that tends to develop more severe influenza complications (7). In general, H3N2 influenza strains have been associated with lower antibody responses and decreased vaccine effectiveness even in well-matched years (8, 9). During the 2014–2015 influenza season, vaccine effectiveness in the Northern Hemisphere against H3-specific influenza was estimated to be at 22% (95% confidence interval [95% CI], 5 to 35%) (10). This low effectiveness was attributed both to the low immunogenicity of the H3 vaccine components (11) and to a mismatch of the H3 component with the circulating H3 viruses (6). Despite the limitations of the 2014–2015 influenza vaccine to prevent disease caused by these antigenically drifted strains, a study carried out in the hospital setting suggested that vaccination could have prevented a more severe disease requiring hospitalization (12).

The reasons for enhanced susceptibility to severe bacterial disease after influenza infection remain poorly defined. Bacteria like Staphylococcus aureus and Streptococcus pneumoniae, which are the most prominent pathogens involved in bacterial superinfection, are common colonizers of the upper respiratory tract (URT) and make up the URT microbiome with other resident microbes. However, viral infection can disrupt this equilibrium and cause loss of some microbial populations and/or overgrowth of other pathogens, resulting in disease. Disruption of the URT microbiota has been found to be associated with community-acquired pneumonia (13), although it is still unclear whether the microbial changes observed are the cause or the consequence of the viral infection. The protective role of resident microbes has also been studied. For example, S. aureus priming mediates recruitment of M2 alveolar macrophages, which reduce influenza pathogenesis by limiting inflammation in the lungs (14).

One aspect not fully explored is how influenza types and strains impact the microbiota in the respiratory tract and whether vaccination could be protective by either reshaping the microbiota or preventing the virus from disrupting its equilibrium. In this regard, even an unmatched influenza vaccine could prevent severe disease by modulating the respiratory bacterial communities. Recent studies have shown an effect of the live-attenuated influenza vaccine (LAIV) on the microbiota of the nasopharynx (15, 16). Although the microbiota of the respiratory tract has been described as the gatekeeper of respiratory health (17), information on how it changes in influenza infection and how it could be impacted by vaccination is still sparse. To address these questions, we characterized the microbiota of the nasopharynx by analyzing samples from individuals diagnosed with influenza A virus (IAV) H3N2 or influenza B virus (IBV) (Yamagata or Victoria) infections during the 2014–2015 influenza season in New York. Investigating in more detail the relationship between host factors, such as age or vaccination status, and the respiratory microbiome in a specific influenza season could help us better identify factors that contribute to influenza disease severity.

## RESULTS

We analyzed a total of 226 nasopharyngeal (NP) swabs from 215 patients diagnosed with IAV (*n* = 157) or IBV (*n* = 58) collected at New York Presbyterian Hospital/Weill Cornell Medicine in New York City during the 2014–2015 influenza season. One patient (patient ID 213 in Table S1 in the supplemental material) was diagnosed with IAV first and then with IBV a month later. Clinical characteristics of individual subjects and influenza vaccination history, including whether the patient had been vaccinated in previous seasons, in the current season (2014-2015), or both, is summarized in Table S1. Of individuals infected with IAV, 43% (67/157) had been vaccinated in the current season, while 62% (97/157) had received the influenza vaccine in one or more of the five previous seasons (Table 1). Of individuals infected with IBV, 41% (24/59) were vaccinated in the current season and 53% (31/59) at some point in the last five seasons (Table 2).

**TABLE 1:** Characteristics of subjects with influenza A included in the study^*a*^

Patient characteristic	No. of patients (%) or parameter value			
	Overall	Young (<18 yo)	Adult (18−64 yo)	Elderly (≥65 yo)
Total no.	157	31	60	66
Age, yr [median (IQR)]	60 (24−77)	3 (0−7)	46 (33−55)	79 (72−86)
Male gender	60 (40)	15 (48)	20 (33)	25 (38)

Care setting				
ED	32 (20)	14 (45)	12 (60)	6 (9)
Inpatient	71 (45)	8 (26)	17 (28)	46 (70)
Outpatient/clinic	54 (34)	9 (29)	31 (52)	14 (21)

Immunocompromised^*b*^				
Yes	40 (25)	4 (13)	19 (32)	17 (26)
No	104 (66)	24 (77)	34 (57)	46 (70)
Unknown	13 (8)	3 (10)	7 (12)	3 (5)

Documented LRTI^*c*^				
Yes	15 (10)	1 (3)	4 (7)	10 (15)
No	126 (80)	26 (84)	47 (78)	53 (80)
Unknown	16 (10)	4 (13)	9 (14)	3 (5)

Vaccination in the current season (2014−2015)				
Yes	67 (43)	11 (35)	23 (38)	33 (50)
No	64 (41)	18 (58)	30 (50)	16 (24)
Unknown	26 (16)	2 (6)	7 (12)	17 (26)

Vaccination in the past 5 yrs				
Yes	97 (62)	14 (45)	38 (63)	45 (68)
No	45 (29)	16 (52)	15 (25)	14 (21)
Unknown	15 (9)	1 (3)	7 (12)	7 (11)

Disposition/outcome				
Home	135 (86)	28 (90)	49 (82)	58 (88)
Hospitalized	5 (3)	0	2 (3)	3 (5)
Death	3 (2)	0	0	3 (5)
Unknown	14 (9)	3 (10)	9 (15)	2 (3)

a Abbreviations: yo, years old; IQR, interquartile range; ED, emergency department; LRTI, lower respiratory tract infection.

b Includes neutropenia, leukemia, lymphoma, stem cell transplant, solid organ transplant, pregnancy, HIV, use of high-dose steroids, monoclonal antibodies, or systemic chemotherapy.

c Diagnosed by chest X-ray.

**TABLE 2:** Characteristics of subjects with influenza B included in the study^*a*^

Patient characteristic	No. of patients (%) or parameter value			
	Overall	Young (<18 yo)	Adult (18−64 yo)	Elderly (≥65 yo)
Total no.	58	8	28	22
Age, yr [median (IQR)]	59 (42−71)	2.5 (1−8)	51 (43−61)	75 (69−88)
Male gender	30 (51)	4 (57)	15 (50)	10 (50)

Care setting				
ED	8 (14)	1 (12)	4 (14)	3 (14)
Inpatient	20 (34)	3 (38)	3 (11)	14 (64)
Outpatient	30 (52)	4 (50)	21 (75)	5 (23)

Immunocompromised^*b*^				
Yes	10 (17)	1 (12)	6 (21)	3 (14)
No	42 (72)	7 (87)	18 (64)	17 (77)
Unknown	6 (10)	0	4 (14)	2 (9)
				
Documented LRTI^*c*^				
Yes	6 (10)	0	1 (4)	5 (21)
No	51 (88)	8 (100)	24 (92)	19 (79)
Unknown	1 (2)	0	1 (4)	0

Vaccination in the current season (2014−2015)				
Yes	24 (41)	4 (50)	7 (25)	13 (59)
No	13 (22)	4 (50)	7 (25)	2 (9)
Unknown	21 (36)	0	14 (50)	7 (32)

Vaccination in the past 5 yrs^*d*^				
Yes	31 (53)	5 (63)	7 (25)	19 (86)
No	7 (12)	2 (25)	5 (18)	0
Unknown	20 (34)	1 (12)	16 (57)	3 (14)

Disposition/outcome				
Home	57 (98)	8 (100)	28 (100)	21 (95)
Death	1 (2)	0	0	1 (5)

a Abbreviations: yo, years old; IQR, interquartile range; ED, emergency department; LRTI, lower respiratory tract infection.

b Includes neutropenia, leukemia, lymphoma, stem cell transplant, solid organ transplant, pregnancy, HIV, use of high-dose steroids, monoclonal antibodies, or systemic chemotherapy.

c Diagnosed by chest X ray.

d Children too young to receive flu vaccine (<1 year old) are not counted.

10.1128/mBio.01296-19.1 TABLE S1 Clinical and sequencing information of single samples included in the study. Download Table S1, XLSX file, 0.05 MB. Copyright © 2019 Ding et al. 2019 Ding et al. This content is distributed under the terms of the Creative Commons Attribution 4.0 International license.

### The microbiota of the nasopharynx is significantly different in influenza virus-infected subjects compared to the microbiota of uninfected individuals.

**FIG 1:**
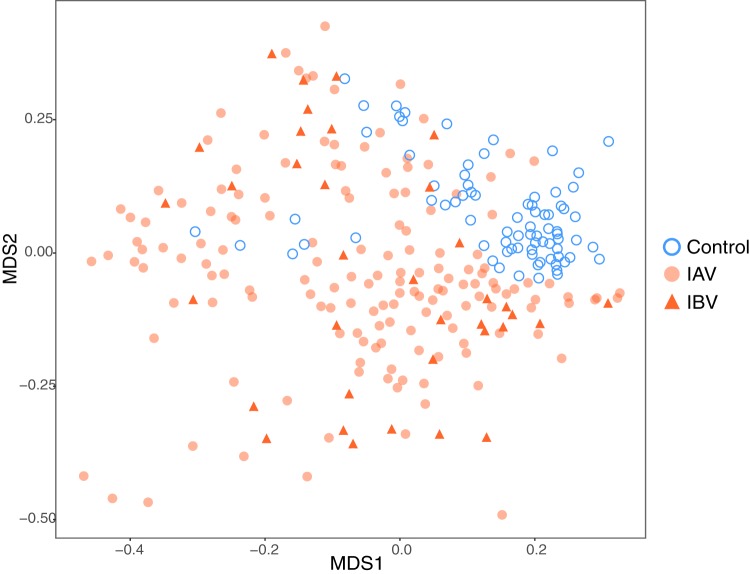
Clustering of influenza virus-infected samples and healthy control samples based on genus-level taxonomic assignments. Clustering is displayed as a nonmetric multidimensional scaling (NMDS) plot of all the samples, in which the dissimilarity between samples is calculated as the Bray-Curtis distance.

**FIG 2:**
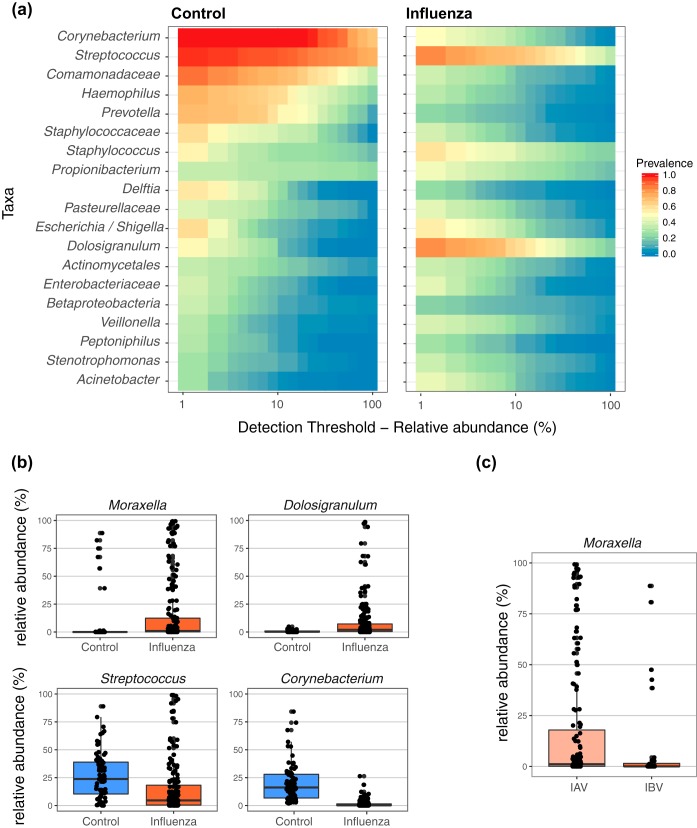
(a) Core microbiota heatmaps showing abundance of taxa and prevalence across samples from healthy controls and influenza virus-infected individuals. Taxa listed were selected on the basis on their prevalence in the two groups of samples. (b) Relative abundance of significant taxa enriched in influenza infection (top graphs) or in healthy controls (bottom graphs). Significance was determined by LEfSe. Whiskers represent values outside the upper and lower quartiles. (c) Relative abundance of significant taxa enriched in influenza A virus infection compared to influenza B virus infection. Significance was determined by LEfSe. Whiskers represent values outside the upper and lower quartiles.

To determine whether the microbial community of the nasopharynx was different in the context of influenza infection, we compared NP swabs collected from influenza virus-infected patients to those from 40 healthy individuals (controls). Although no significant difference in microbial alpha diversity was detected between the influenza and control groups (Shannon index and inverse Simpson index were tested, *P* > 0.05), the microbial compositions were significantly different (*P* value < 0.001 by analysis of molecular variance [AMOVA] test), leading to samples clustering within each group, as visualized by multidimensional scaling (Fig. 1). We compared the relative abundance and prevalence of taxa that were represented in both groups, i.e., the core microbiota (Fig. 2a), and identified signature taxa for each group using linear discriminant analysis and effective size comparisons (LEfSe) analysis (Fig. 2b). The control samples appeared to be dominated by *Corynebacterium* and *Streptococcus*, while the influenza virus-infected individuals had a slightly lower prevalence and abundance of *Streptococcus*, an enrichment of *Dolosigranulum*, and very low prevalence of *Corynebacterium* (Fig. 2a).

We also used LEfSe to identify taxonomic features that were most likely to significantly characterize the influenza-related compositional differences of the microbiota. We observed four taxa (Table S2), including *Moraxella* and *Dolosigranulum*, that were significantly enriched in the influenza group (Fig. 2b, top panel). We identified seven key taxa (Table S2), with the top two being *Streptococcus* and *Corynebacterium*, which had a relative abundance that was significantly higher in the control group (Fig. 2b, bottom panel).

10.1128/mBio.01296-19.2 TABLE S2 Taxa identified by LEfSe as significantly enriched in the influenza virus-infected or control groups. Download Table S2, XLSX file, 0.1 MB. Copyright © 2019 Ding et al. 2019 Ding et al. This content is distributed under the terms of the Creative Commons Attribution 4.0 International license.

Considering that IBV is often associated with milder disease and to determine whether IBV infection was associated with a different microbial profile than IAV infection, we compared the NP microbiota of IAV- and IBV-infected subjects. Moderate differences in their compositions were detected (Fig. 1; *P* value < 0.01 by AMOVA). Using LEfSe, we observed that the relative abundance of four taxa, including *Moraxella* (Fig. 2c) were significantly higher in IAV infections (Table S3), while four taxa were significantly higher in IBV infections (Table S3).

10.1128/mBio.01296-19.3 TABLE S3 Taxa identified by LEfSe as significantly enriched in the influenza A virus- or influenza B virus-infected subjects. Download Table S3, XLSX file, 0.01 MB. Copyright © 2019 Ding et al. 2019 Ding et al. This content is distributed under the terms of the Creative Commons Attribution 4.0 International license.

**FIG 3:**
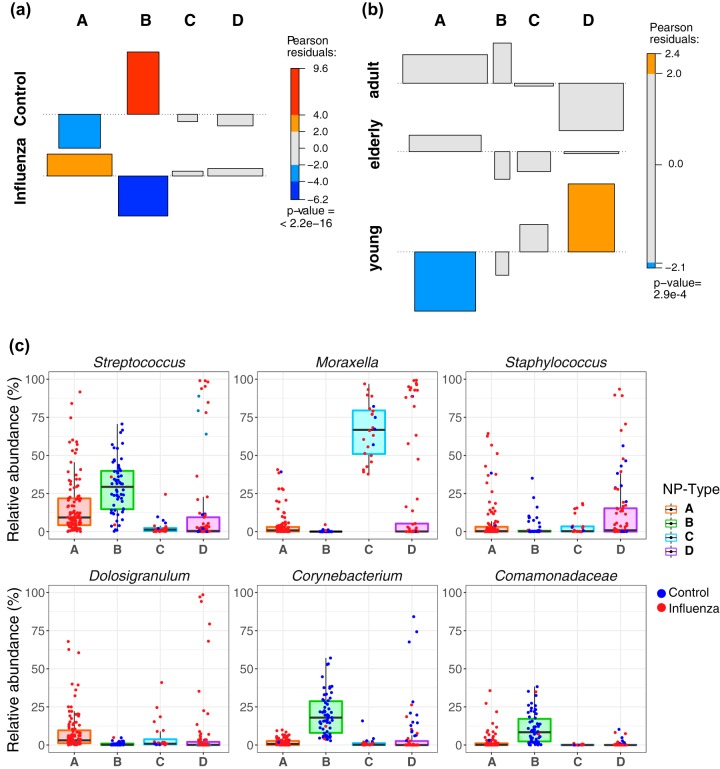
(a) Association between the four nasopharyngeal (NP) types and influenza infection status, determined by chi-square test. (b) Association between the four NP types and the three age groups in influenza virus-infected subjects. (c) Relative abundance of the dominant microbial taxa in each NP type.

We further explored the association between influenza infection and microbial community structure. To do so, we partitioned the data into community types using Dirichlet multinomial mixture models. We identified four microbial community types (NP types) in the subjects tested. NP type A was significantly enriched in influenza infection, while NP type B dominated in the control group (Fig. 3a and Table 3). While both community types C and D were slightly enriched in influenza patients, it was not at a significant level. To study the effect of age on the core microbiome of influenza virus-infected subjects, we divided them in three groups: young (<18 years), adult (18 to 64 years), and elderly (65 years or older). In the influenza virus-infected subjects only, the less common influenza-associated NP type D was found predominantly in the young (<18 years old) (Fig. 3b), while NP type A was underrepresented in this group. We analyzed the abundance of the dominant taxa in each NP type (Fig. 3c). NP type A was comprised primarily of *Streptococcus* and *Dolosigranulum*; NP type B, enriched in the control cohort, was dominated by a combination of *Streptococcus*, *Corynebacterium*, and *Comamonadaceae*. NP type C was dominated by *Moraxella*, while NP type D had an overrepresentation of *Staphylococcus*. The different communities did not associate differently with either of the flu types (IAV versus IBV).

**TABLE 3:** Samples associated with each community type

Patient group	No. of samples associated with the following community type:			
	NP type A	NP type B	NP type C	NP type D
Healthy	2	59	4	14
Infected	105	3	20	63

### Association of vaccination with the nasopharynx microbiota is different in IAV and IBV.

**FIG 4:**
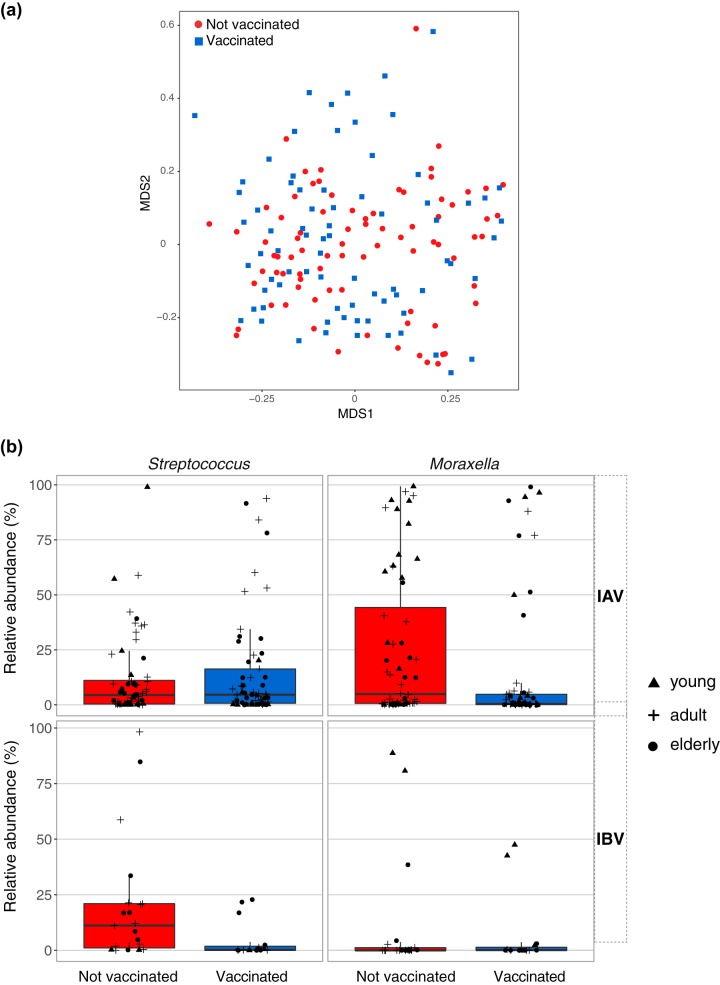
(a) Beta diversity ordination calculated by NMDS using the Bray-Curtis dissimilarity of samples from vaccinated and unvaccinated individuals. (b) Relative abundance of *Streptococcus* and *Moraxella* in unvaccinated patients (left) and vaccinated patients (right), separated by IAV (top) and IBV infections (bottom). The age groups of the individuals from which the samples were collected are indicated by different symbols.

We determined whether vaccination in the current season had an association with the microbial composition of the NP in the influenza virus-infected individuals. We did not observe differences in sample clustering between individuals who were vaccinated or not vaccinated, indicating that microbial composition was similar in both groups (Fig. 4a; *P* value of >0.01 in AMOVA). However, when looking for taxonomic features that significantly characterized each group, we observe by LEfSe analysis an enrichment of specific taxa in the unvaccinated subjects—*Moraxella* in IAV (*P* = 0.002) and *Streptococcus* in IBV (*P* = 0.010) (Fig. 4b).

**FIG 5:**
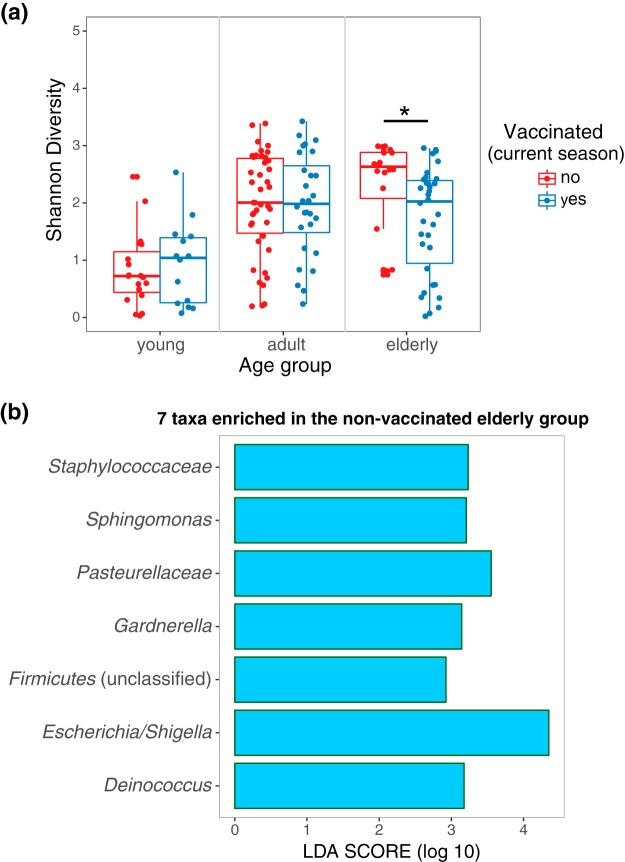
(a) Comparison of alpha diversities (calculated as Shannon index) of unvaccinated patients with those of vaccinated patients, separated by age groups. (b) Linear discriminant analysis (LDA) score of the seven microbes found to be significantly enriched in unvaccinated elderly patients against vaccinated elderly patients, determined by LEfSe.

We also tested for age-dependent differences in the microbiota of the nasopharynx in vaccinated and unvaccinated individuals infected with either IAV or IBV, but we did not observe any significant differences in enriched taxa between age groups. However, when testing overall microbial diversity (as measured by Shannon entropy), we observe higher microbial diversity in the unvaccinated elderly than in the vaccinated elderly (*P* value = 0.005 by Wilcox test) (Fig. 5a); we did not observe a similar effect in the two other age groups. To further study what compositional differences contributed to this age-specific difference in microbial diversity, by using LEfSe we identified seven microbial taxa for which relative abundance was significantly higher in the unvaccinated elderly group (Fig. 5b). Some members of these taxa such as *Staphylococcaceae*, Gram-negative bacteria (*Pasteurellaceae* and *Escherichia* or *Shigella*), and *Sphingomonas* also include species known to be associated with postinfluenza (including pneumonia) and nosocomial infections (18). Finally, we tested whether any of the clinical variables (listed in Table 1 and Table S1), including pneumonia, antibiotic usage, Tamiflu usage, and immune status (immunocompromised), was associated with specific features of the nasopharyngeal microbiota, but we did not find any significant association.

### Influenza virus genetic diversity affects the microbiota of the nasopharynx.

**FIG 6:**
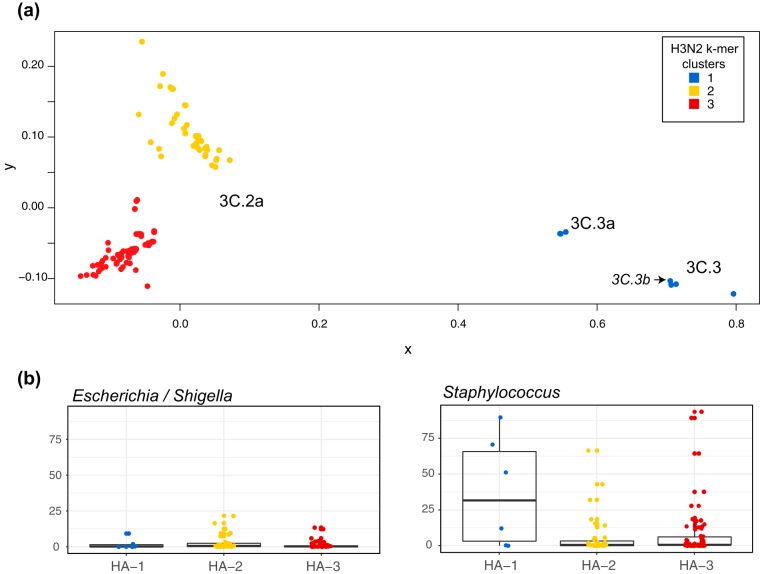
(a) Clustering of IAV patients based on the genetic diversity of the HA segment. (b) Relative abundance of the microbes that vary across the HA genetic clusters.

While we observed differences in microbial enrichment between individuals infected with IAV and IBV, and in community types for infected versus control individuals, we explored whether there was also an association between IAV genetic diversity and the microbial community within the nasopharynx. We first performed a k-mer analysis to identify underlying influenza virus sequence signatures for each sample and compared them to each other, visualizing this measure of genetic distance by multidimensional scaling (Fig. 6a). Three clusters were identified for influenza A/H3N2, with two clusters corresponding to the 3C.2a genetic clade. While we did not see a correspondence between the sample clustering profile and the NP type microbial profiles (data not shown), we did observe by performing LEfSe analysis that one of the two 3C.2a clusters, HA-2, had a significantly higher relative abundance of *Escherichia* (*Shigella*) (Fig. 6b) compared to the other 3C.2a cluster (HA-3) and the 3C.3 cluster (HA-1). While all three clusters had samples with high relative abundance of *Staphylococcus*, it was significantly different across the three hemagglutinin (HA) groups, with potentially higher relative abundance in the group corresponding to clade 3C.3 (HA-1).

## DISCUSSION

A number of studies have looked at the respiratory tract microbiota and influenza infections (15, 19–21), but this is the first to explore the respiratory microbiota in both IAV H3N2 and IBV infection in the context of vaccination in a year with low vaccine effectiveness. One study characterized the microbiota in patients infected with the 2009 pandemic H1N1 influenza. They used a *cpn60* amplicon sequencing method and no healthy controls were involved, so no inference was made regarding the alteration of the microbiota caused by influenza infection (19). We have shown that IAV and IBV virus infections were associated with a significantly different microbial community profile than that for uninfected individuals. The microbiota of the nasopharynx in infected individuals was enriched with taxa such as *Dolosigranulum* and *Staphylococcus* compared to the microbiota of the control group. These two genera have previously been shown to be associated with pneumonia (22, 23). *Dolosigranulum* microbes are Gram-positive bacteria for which currently only one species has been identified (Dolosigranulum pigrum). Although a rare opportunistic pathogen, D. pigrum has been confirmed as a causative agent in different types of pneumonia and septicemia (22, 23). The significant enrichment of these types of Gram-positive bacteria compared to healthy controls indicates that the predisposition to superinfection could be initiated early on in the infection, likely due in part to the dysregulation of the innate and immune response by the virus (reviewed in reference 23). We also see a significantly higher relative abundance of *Moraxella* spp., which are Gram-negative bacteria, in influenza virus-infected subjects compared to the healthy control group. A number of species in this genus are resident microbes of mucosal surfaces, occasionally leading to opportunistic infections. *Moraxella* spp. have already been recognized as human respiratory tract pathogens (24) and have been observed in some cases to lead to influenza infection complications (25). Interestingly, an increased prevalence of *Moraxella* spp. has been reported in individuals with acute viral upper respiratory infections caused by viruses (26).

In the analysis of community types, we saw that one type in particular (NP type A) was enriched in influenza virus-infected subjects and was comprised primarily of *Dolosigranulum* and *Streptococcus*. We also identified a negative association between influenza infection and *Corynebacterium*. *Corynebacterium* has been found to commonly colonize the human nose and skin and was shown to be overrepresented in children free of Streptococcus pneumoniae (27). Corynebacterium accolens was shown to inhibit the growth of S. pneumoniae by releasing antibacterial free fatty acids (27). A negative correlation between S. aureus and *Corynebacterium* abundance was also previously observed (28) with a recent study showing that *Corynebacterium* inhibits the virulence of S. aureus (29). In our own data, we also observed that *Staphylococcus* was present at very low relative abundance in NP type B, which was enriched in the control group and dominated by a combination of *Streptococcus*, *Corynebacterium*, and *Comamonadaceae*. Because species-level resolution for taxonomic assignment is difficult with 16S rRNA gene sequence analysis, we do not know whether S. pneumoniae was the dominant species for the *Streptococcus* identified in both NP type A and NP type B. Overall, these observations suggest that *Corynebacterium* could potentially protect the respiratory tract from pathogenic bacteria such as S. aureus and S. pneumoniae that are the most common cause of postinfluenza pneumonia. More work is needed to confirm that the observed lack of *Corynebacterium* in the nasopharynges of influenza virus-infected individuals contributes to the increased likelihood of influenza-induced pneumonia.

Since our study was cross-sectional and we studied the composition of the nasopharynx microbiota at diagnosis, we cannot determine whether differences between healthy controls and influenza virus-infected individuals are due to the infection, the presence of a microbial community that predisposes to infection, or a combination of both. A longitudinal study of sputum samples from rhinovirus-infected individuals showed a rise in bacterial burden with a higher prevalence of Haemophilus influenzae associated with infection (30). Longitudinal influenza infection studies have reported conflicting results with one study showing that the administration of live-attenuated influenza virus (LAIV) can modify the microbiota of the nasal cavity (16), while a study where volunteers challenged with an H3N2 strain were sampled over a 30-day period did not show any changes in the oropharyngeal microbiota (21). This lack of an effect may be due to the fact that the cohort was comprised of young and healthy volunteers with many who developed very mild disease (19 out the 52 challenged individuals). In contrast, our cohort includes patients from all age groups, including young and elderly, with a range of disease severity. A recent household transmission study shows that influenza susceptibility is associated with differences in the overall bacterial community structure, with a particularly increased influenza risk in young children (20). These differences between studies suggest that patient characteristics such as age, comorbidities, vaccination status, treatments, and viral characteristics need to be considered when studying the effect of influenza infection on the respiratory microbiota.

Our study also addresses the association of vaccination with differences in the microbiota of the nasopharynx during influenza infection. The effectiveness of the influenza vaccine varies in different seasons (31, 32) due to a number of factors, such as vaccine strain mismatch and host immune status, including history of previous influenza vaccination. We suggest that another potential factor is the host microbiome. Recent studies have shown that the human microbiota, by impacting immune cell development and differentiation, could influence adjuvant and vaccine efficacy (33). LAIV was shown to affect the microbiota of the nasopharynx (15, 16) and lead to an increased abundance of specific microbes associated with IgA responses (15). A study on the effects of trivalent LAIV on bacterial carriage in the nasopharynges of toddlers showed that there was an increase in S. pneumoniae and Moraxella catarrhalis density 28 days after vaccination (34), indicating that the influenza virus, even when attenuated, could impact carriage density.

We show that in influenza virus-infected individuals the lack of vaccination in the current season is associated with the enrichment of different microbial taxa, such as *Moraxella* and *Streptococcus*, depending on the type of influenza virus (IAV versus IBV). Although we cannot exclude or confirm that other confounding factors may also play a role in shaping the nasopharyngeal microbiota of influenza virus-infected patients, we tested for factors that were included in our demographic data, such as age, sex, and antibiotic usage, and did not observe any other significant association. However, an aspect missing is how vaccination can specifically reduce the risk for respiratory comorbidities, which can be largely attributed to the disruption of the microbial community within the respiratory tract (17). Because of an increased risk of infection, young and elderly populations get the most benefit from influenza vaccination (35). We found that in the elderly group (65 years old and older), the microbial diversity in the nasopharynges of unvaccinated patients was significantly higher than in the vaccinated patients, with an overrepresentation of taxa that include pathogenic species associated with nosocomial infections. A recent study has linked increased nasopharynx microbial diversity with pneumonia infections in the elderly population (13). While even an unmatched influenza vaccine can provide some level of cross-protection, our findings suggest that a protective effect could also be mediated by modifications in the microbiota that can help limit the growth of opportunistic pathogens.

### Conclusion.

Our aims in this study were to determine whether the microbiota of the nasopharynx was different in individuals with influenza infection and to identify factors associated with the variations observed between infected subjects in different age groups. We found that during influenza infection, the nasopharyngeal microbiota of vaccinated individuals was strongly associated with higher levels of specific microbial taxa, with different microbial profiles relative to virus types and clades. These observations provide new insight into influenza infection and highlight a need for more studies to explore the mechanism of how influenza vaccines—live attenuated or killed—interact with the respiratory microbiota.

## MATERIALS AND METHODS

### Subjects and sample acquisition.

Nasopharyngeal (NP) swabs collected from subjects of any age and sex that were sent to the New York Presbyterian Hospital microbiology laboratory for influenza testing in the 2014–2015 season were used for this study. All samples were confirmed by Film Array (Biofire) to be either IAV H3N2 or IBV positive. Clinical data were abstracted from the electronic medical record. For every subject, we collected data on patient demographics, comorbidities and related treatments, influenza vaccination history, underlying malignancy status and treatments, antibiotics and antiviral treatments, clinical course, including infections and therapies, and microbiology data. We also collected 80 NP swabs from 40 healthy patients living in New York City as controls. These individuals were enrolled as part of an IRB-approved study aiming to characterize the respiratory microbiome in immunocompromised patients and healthy controls (T. Ding, R. Soave, A. Geber, E. Ghedin, and M. Salvatore, unpublished data) (see Table S4 in the supplemental material). These volunteers represented a mix of hospital clinic workers and community members, as we wanted to establish whether the hospital environment contributed to the microbiota observed since a number of our patients were hospitalized. We did not observe any difference between hospital workers and community members. Total DNA and RNA were extracted from each sample and subjected to 16S rRNA gene sequencing for microbiota profiling and influenza virus gene segment sequencing, respectively.

10.1128/mBio.01296-19.4 TABLE S4 Characteristics of healthy volunteers who provided samples. Download Table S4, XLSX file, 0.01 MB. Copyright © 2019 Ding et al. 2019 Ding et al. This content is distributed under the terms of the Creative Commons Attribution 4.0 International license.

### DNA extraction and 16S rRNA gene sequencing.

DNA extractions from the NP swab specimens were performed using the PowerSoil DNA Isolation kit (MO BIO Laboratories Inc.) in a sterilized class II type A2 biological safety level 2 cabinet (Labgard ES Air, NuAire). The swabs were processed in batches, and the cotton tip of each swab was cut off and transferred into the PowerBead tubes as the starting material. Nuclease-free water (Ambion, ThermoFisher Scientific Inc.) was also processed by the same DNA extraction procedure as the specimens and the healthy individual specimen control. Extracted DNA was eluted in 50 μl nuclease-free water and stored at –20°C until processing. Extracted DNA was then used in a PCR to amplify the V4 hypervariable region of the 16S rRNA gene using primer pair 515F/806R to prepare the sequencing library (36). Six microliters of extracted DNA from swab samples was used as the template in a final volume of 25 μl, with 0.35 μl Q5 Hot Start High-Fidelity DNA polymerase (New England BioLabs Inc.), 5 μl of 5× Q5 buffer, 0.5 μl dNTP mix, and 0.5 μM forward and reverse primers. Thermal cycling conditions were as follows: (i) 94°C for 2 min; (ii) 33 cycles, with 1 cycle consisting of 94°C for 30 s, 55°C for 30 s, and 72°C for 90 s; (iii) 72°C for 10 min. PCR products were purified using 0.65× volumes of AMPure XP beads (Beckman Coulter) and eluted into 20 μl low TE (10 mM Tris, 0.1 mM EDTA [pH 8.0]) on the Bravo Automated Liquid Handling Platform (Agilent Technologies). Eluted PCR products were quantified with a Quant-iT double-stranded DNA (ds-DNA) High-Sensitivity assay kit (Invitrogen) on an Infinite M200 plate reader (Tecan) according to the manufacturer’s instructions and were combined with equal input mass into a sequencing pool. The pool was then purified again with 0.65× volumes of AMPure XP beads and analyzed on a 2200 TapeStation (Agilent Technologies) using a High Sensitivity D1000 ScreenTape (Agilent Technologies) to confirm the integrity of the sequencing library. Finally, the sequencing pool was quantified by qPCR using the KAPA Library Quantification kit (KAPA Biosystems) on a Roche 480 LightCycler. The library was sequenced at the Genomics Core Facility of the Center for Genomics and Systems Biology, New York University, using an Illumina PE 2x250 V2 kit on an Illumina MiSeq sequencer.

### 16S rRNA gene sequence analysis.

The sequencing data were processed using the 16S rRNA gene sequence curation pipeline that was implemented in the mothur software package (37) following a previously described procedure (38). Briefly, the raw sequences as fastq files were extracted from sff files, and any sequence that had mismatches to the barcode, more than one mismatch to the primers, more than eight nucleotide homopolymers, or ambiguous base calls was removed. Trimmed sequences were denoised using PyroNoise (39) and then aligned against a customized SILVA database (40). Chimeric sequences were detected and removed using a *de novo* Uchime algorithm that was implemented in mothur (41). The dechimeric sequences were classified using the naive Bayesian Classifier trained against a customized version of the RDP training set (v9). A minimum classification score of 80% was required, and 1,000 pseudobootstrap iterations were used. The taxonomy of the remaining sequences was used to assign the sequences to genus-level phylotypes, also known as operational taxonomic units (OTU), and this allowed us to make a table of counts for the number of times each phylotype was observed in each sample. Phylotypes that were identified in less than 20% of the total samples were removed from subsequent analysis. Samples with fewer than 1,000 reads were removed from downstream analysis, and all samples were subsampled or rarified to 1,000 reads to perform subsequent analyses. Signature microbial groups were identified by performing LEfSe (linear discriminant analysis effective size) analysis (41) implemented in mothur. Bacterial community types were defined using a Dirichlet multinomial mixture (DMM) algorithm-based method that was previously described and implemented in mothur (42). Statistical tests, including Wilcoxon signed rank test, chi-square test, and Student’s *t* test were performed in R.

### RNA extraction and viral segment sequencing.

Total RNA was extracted from each sample according to the manufacturer’s recommendations using 100 μl of Viral Transport Media as input for the RNeasy Micro kit (Qiagen). Influenza genomic RNA was subsequently converted into cDNA and amplified (40 cycles of PCR) via the SuperScript III One-Step RT-PCR System with Platinum *Taq* High-Fidelity DNA polymerase (Invitrogen) according to previously published methods (43, 44). Each successfully amplified influenza RNA sample was prepared for sequencing by one of two methods. Concurrent experimental work confirmed that samples prepared by both methods yielded identical minor variant profiles. Sixty-five of the samples used for subsequent analyses were sonicated in a microTUBE (Covaris) using the S220 Focused-ultrasonicator (Covaris). The fragmented cDNA was purified by 0.8× volumes AMPure XP beads on the Agilent Bravo and quantified by using the Quant-iT High-Sensitivity dsDNA assay kit. Fifty nanograms of cDNA from each sample were used an input for the NEBNext Ultra DNA Library Prep kit for Illumina (New England Biolabs) according to the manufacturer’s recommendations. The remaining cDNA samples were prepared for sequencing using a modified version of the Nextera DNA Library Preparation kit protocol (Illumina). Amplicons were purified by 0.8× volumes AMPure XP beads on the Agilent Bravo and quantified via the Quant-iT High-Sensitivity dsDNA assay kit before normalization to a constant concentration (2.5 ng/μl); 2.5 ng of cDNA from each sample was used as input for Nextera library preparation. Individual libraries prepared by either method were quantified via the Quant-iT High-Sensitivity dsDNA assay kit and pooled with equal input mass before repurification and size adjustment with 0.6× volumes AMPure XP beads. Each of the three resultant pools (one prepared with NEBNext, two prepared with Nextera) was quantified by qPCR using the KAPA Library Quantification kit on a Roche 480 LightCycler, and its size distribution was measured on a 2200 TapeStation using a D1000 ScreenTape (Agilent Technologies). Each pool was sequenced at the Genomics Core Facility at the Center for Genomics and Systems Biology, New York University using an Illumina PE 2x250 V2 kit on an Illumina MiSeq Sequencer. Each pool was seeded at 12 pM and included a 10% PhiX spike-in to compensate for potential low base diversity.

### Viral sequencing data analysis.

Samples were trimmed using Trimmomatic, and the trimmed reads of each sample were mapped using the Burrows-Wheeler Alignment Tool (bwa) (45) with default parameters against the A/New York/03/2015 H3N2 strain for IAV H3N2-infected samples and against both B/Kentucky/28/2015 (Victoria) and B/New York/WC-LVD-15-007/2015 (Yamagata) strains for IBV-infected samples. These samples were then processed for quality filtering and analysis with samtools (46). The average quality of any given read had to pass a phred score of 25. For generation of consensus sequences, any given site had to be covered by at least 200 reads. Minority variants were discovered by using statistical tests to minimize false-positive single nucleotide variant (SNV) calls that can be caused by sequence-specific errors. This involves using a binomial test to ensure that reads come from both the forward and reverse orientation. Additional thresholds for minority variant detection include a frequency of 1%, which accounts for the sequencing noise as determined by a plasmid control sample, and a coverage of 500×.

### Ethics approval.

The study was approved by the Weill Cornell Medical College Institutional Review Board and the New York University Institutional Review Board (Weill Cornell Medicine IRB protocol 1506016280).

### Data availability.

The sequencing data from this study are available in the Sequence Read Archive (SRA) under the following accession numbers: SRP132207 for 16S rRNA genes and PRJNA431639 for influenza virus data.
